# Predicting Kidney Failure, Cardiovascular Disease and Death in Advanced CKD Patients

**DOI:** 10.1016/j.ekir.2022.07.165

**Published:** 2022-08-02

**Authors:** Chava L. Ramspek, Rosemarijn Boekee, Marie Evans, Olof Heimburger, Charlotte M. Snead, Fergus J. Caskey, Claudia Torino, Gaetana Porto, Maciej Szymczak, Magdalena Krajewska, Christiane Drechsler, Christoph Wanner, Nicholas C. Chesnaye, Kitty J. Jager, Friedo W. Dekker, Maarten G.J. Snoeijs, Joris I. Rotmans, Merel van Diepen, EQUAL study investigators, Adamasco Cupisti, Adelia Sagliocca, Alberto Ferraro, Aleksandra Musiała, Alessandra Mele, Alessandro Naticchia, Alex Còsaro, Alistair Woodman, Andrea Ranghino, Andrea Stucchi, Andreas Jonsson, Andreas Schneider, Angelo Pignataro, Anita Schrander, Anke Torp, Anna McKeever, Anna Szymczak, Anna-Lena Blom, Antonella De Blasio, Antonello Pani, Aris Tsalouichos, Asad Ullah, Barbara McLaren, Bastiaan van Dam, Beate Iwig, Bellasi Antonio, Biagio Raffaele Di Iorio, Björn Rogland, Boris Perras, Butti Alessandra, Camille Harron, Carin Wallquist, Carl Siegert, Carla Barrett, Carlo Gaillard, Carlo Garofalo, Cataldo Abaterusso, Charles Beerenhout, Charlotte O'Toole, Chiara Somma, Christian Marx, Christina Summersgill, Christof Blaser, Claudia D'alessandro, Claudia Emde, Claudia Zullo, Claudio Pozzi, Colin Geddes, Cornelis Verburgh, Daniela Bergamo, Daniele Ciurlino, Daria Motta, Deborah Glowski, Deborah McGlynn, Denes Vargas, Detlef Krieter, Domenico Russo, Dunja Fuchs, Dympna Sands, Ellen Hoogeveen, Ellen Irmler, Emöke Dimény, Enrico Favaro, Eva Platen, Ewelina Olczyk, Ewout Hoorn, Federica Vigotti, Ferruccio Ansali, Ferruccio Conte, Francesca Cianciotta, Francesca Giacchino, Francesco Cappellaio, Francesco Pizzarelli, Fredrik Sundelin, Fredrik Uhlin, Gaetano Greco, Geena Roy, Gaetana Porto, Giada Bigatti, Giancarlo Marinangeli, Gianfranca Cabiddu, Gillian Hirst, Giordano Fumagalli, Giorgia Caloro, Giorgina Piccoli, Giovanbattista Capasso, Giovanni Gambaro, Giuliana Tognarelli, Giuseppe Bonforte, Giuseppe Conte, Giuseppe Toscano, Goffredo Del Rosso, Gunilla Welander, Hanna Augustyniak-Bartosik, Hans Boots, Hans Schmidt-Gürtler, Hayley King, Helen McNally, Hendrik Schlee, Henk Boom, Holger Naujoks, Houda Masri-Senghor, Hugh Murtagh, Hugh Rayner, Ilona Miśkowiec-Wiśniewska, Ines Schlee, Irene Capizzi, Isabel Bascaran Hernandez, Ivano Baragetti, Jacek Manitius, Jane Turner, Jan-Willem Eijgenraam, Jeroen Kooman, Joachim Beige, Joanna Pondel, Joanne Wilcox, Jocelyn Berdeprado, Jochen Röthele, Jonathan Wong, Joris Rotmans, Joyce Banda, Justyna Mazur, Kai Hahn, Kamila Jędrzejak, Katarzyna Nowańska, Katja Blouin, Katrin Neumeier, Kirsteen Jones, Kirsten Anding-Rost, Knut-Christian Gröntoft, Lamberto Oldrizzi, Lesley Haydock, Liffert Vogt, Lily Wilkinson, Loreto Gesualdo, Lothar Schramm, Luigi Biancone, Łukasz Nowak, Maarten Raasveld, Magdalena Durlik, Manuela Magnano, Marc Vervloet, Marco Ricardi, Margaret Carmody, Maria Di Bari, Maria Laudato, Maria Luisa Sirico, Maria Stendahl, Maria Svensson, Maria Weetman, Marjolijn van Buren, Martin Joinson, Martina Ferraresi, Mary Dutton, Merel van Diepen, Michael Matthews, Michele Provenzano, Monika Hopf, Moreno Malaguti, Nadja Wuttke, Neal Morgan, Nicola Palmieri, Nikolaus Frischmuth, Nina Bleakley, Paola Murrone, Paul Cockwell, Paul Leurs, Paul Roderick, Pauline Voskamp, Pavlos Kashioulis, Pawlos Ichtiaris, Peter Blankestijn, Petra Kirste, Petra Schulz, Phil Mason, Philip Kalra, Pietro Cirillo, Pietro Dattolo, Pina Acampora, Rincy Sajith, Rita Nigro, Roberto Boero, Roberto Scarpioni, Rosa Sicoli, Rosella Malandra, Sabine Aign, Sabine Cäsar, Sadie van Esch, Sally Chapman, Sandra Biribauer, Santee Navjee, Sarah Crosbie, Sharon Brown, Sheila Tickle, Sherin Manan, Silke Röser, Silvana Savoldi, Silvio Bertoli, Silvio Borrelli, Siska Boorsma, Stefan Heidenreich, Stefan Melander, Stefania Maxia, Stefano Maffei, Stefano Mangano, Stephanie Palm, Stijn Konings, Suresh Mathavakkannan, Susanne Schwedler, Sylke Delrieux, Sylvia Renker, Sylvia Schättel, Szyszkowska Dorota, Teresa Cicchetti, Teresa Nieszporek, Theresa Stephan, Thomas Schmiedeke, Thomas Weinreich, Til Leimbach, Tiziana Rappa, Tora Almquist, Torsten Stövesand, Udo Bahner, Ulrika Jensen, Valentina Palazzo, Walter De Simone, Wolfgang Seeger, Ying Kuan, Zbigniew Heleniak, Zeynep Aydin

**Affiliations:** 1 Department of Clinical Epidemiology, Leiden University Medical Center, Leiden, the Netherlands; 2 Division of Renal Medicine, Department of Clinical Science, Intervention and Technology, Karolinska Institute and Karolinska University Hospital, Stockholm, Sweden; 3 Department of Population Health Sciences, Bristol Medical School, University of Bristol, Bristol, UK; 4 Department of Clinical Epidemiology of Renal Diseases and Hypertension, Consiglio Nazionale della Ricerche-Istituto di Fisiologia Clinica, Reggio Calabria, Italy; 5 Grande Ospedale Metropolitano, Bianchi Melacrino Morelli, Reggio Calabria, Italy; 6 Department of Nephrology and Transplantation Medicine, Wroclaw Medical University, Wroclaw, Poland; 7 Division of Nephrology, Department of Internal Medicine, University Hospital Wurzburg, Wurzburg, Germany; 8 Division of Nephrology, University Hospital of Wurzburg, Wurzburg, Germany; 9 Department of Medical Informatics, Academic Medical Center, University of Amsterdam, Amsterdam Public Health Research Institute, Amsterdam, the Netherlands; 10 Department of Vascular Surgery, Maastricht University Medical Center, Maastricht, the Netherlands; 11 Department of Internal Medicine, Leiden University Medical Center, Leiden, the Netherlands

**Keywords:** CKD, cardiovascular disease, death, external validation, kidney failure, prognostic model

## Abstract

**Introduction:**

Predicting the timing and occurrence of kidney replacement therapy (KRT), cardiovascular events, and death among patients with advanced chronic kidney disease (CKD) is clinically useful and relevant. We aimed to externally validate a recently developed CKD G4+ risk calculator for these outcomes and to assess its potential clinical impact in guiding vascular access placement.

**Methods:**

We included 1517 patients from the European Quality (EQUAL) study, a European multicentre prospective cohort study of nephrology-referred advanced CKD patients aged ≥65 years. Model performance was assessed based on discrimination and calibration. Potential clinical utility for timing of referral for vascular access placement was studied with diagnostic measures and decision curve analysis (DCA).

**Results:**

The model showed a good discrimination for KRT and “death after KRT,” with 2-year concordance (C) statistics of 0.74 and 0.76, respectively. Discrimination for cardiovascular events (2-year C-statistic: 0.70) and overall death (2-year C-statistic: 0.61) was poorer. Calibration was fairly accurate. Decision curves illustrated that using the model to guide vascular access referral would generally lead to less unused arteriovenous fistulas (AVFs) than following estimated glomerular filtration rate (eGFR) thresholds.

**Conclusion:**

This study shows moderate to good predictive performance of the model in an older cohort of nephrology-referred patients with advanced CKD. Using the model to guide referral for vascular access placement has potential in combating unnecessary vascular surgeries.

## Graphical abstract

**Figure:**
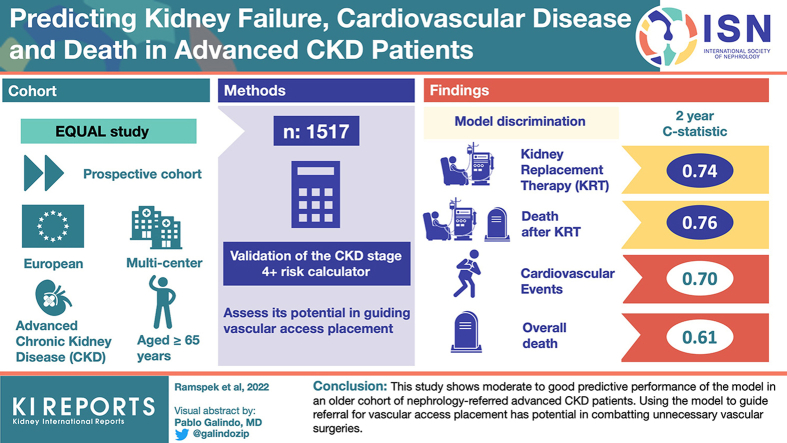

See Commentary on Page 2122

CKD progression rates vary greatly between individuals, which complicates decision making about disease management.^1^^,^^2^ In recent years, multiple prognostic models have been developed in order to provide individualized absolute risks for kidney failure.^3^ When using these prediction models in patients with advanced CKD, it is important to consider that many patients will never reach kidney failure due to death from other causes (competing events). This is particularly the case for older individuals with slow progression or serious comorbidity.^4^ Unfortunately, most existing prediction models fail to account for such competing events, which in turn leads to an overestimation of the risk of kidney failure.^5^ Recently, a promising CKD G4+ risk calculator was developed by Grams *et al.*^6^ (from here on referred to as the Grams model), which predicts the timing and occurrence of not only kidney failure, but also cardiovascular events and death in patients with advanced CKD. By predicting various adverse outcomes in a single model, this tool accounts for competing events and therefore has large potential to improve care for patients with advanced CKD. To our knowledge, the Grams model has only been externally validated for the outcome KRT and death before dialysis and has yet to be validated for cardiovascular disease (CVD) and overall death.^4^^,^^7^^,^^8^ Before use, external validation of prediction models is crucial in order to determine the accuracy in new patients.

The Grams model could be used in various clinical scenarios, such as discussions on the best KRT modality going forward, if any), and the timely preparation for KRT. A report from the Kidney Disease: Improving Global Outcomes group suggested using the Grams model for timely referral for vascular access placement.^1^ Obtaining a working vascular access in the form of an AVF (generally the preferred access for starting hemodialysis (HD) can take up to several months and timing is vital.^9^^,^^10^ AVF placement that is too early is injudicious due to the potential of an unnecessary surgery and the risk of complications. Approximately 30% of patients who receive an AVF will not initiate dialysis within a 2-year period.^11^^,^^12^ On the other hand, late placement can also be harmful, especially when starting dialysis using a central venous catheter, because this is associated with an increased risk of thoracic central venous obstruction and sepsis.^13^ Timing of AVF referral and placement is currently mainly determined by the physician’s expertise.^9^^,^^14^^,^^15^ The European Society for Vascular Surgery advise that a permanent vascular access should be created 3 to 6 months before HD initiation. Nevertheless, predicting the start of HD is not easy. Although more directive guidelines exist, these are generally based on eGFR and lack individualization.^16^^,^^17^ In their 2019 update, the Kidney Foundation’s Kidney Disease Outcomes Quality Initiative advised assessment for vascular access for patients with a ≥50% risk of KRT within 2 years or an eGFR of ≤15 ml/min per 1.73 m^2^, but added that this advice is solely based on expert opinion as empirical evidence is lacking.^18^

The aim of this study is two fold. First, we aim to externally validate the complete Grams model in a nephrology-referred cohort of patients with advanced CKD. Second, we aim to assess the potential clinical impact of the Grams model for guiding timely KRT preparation, including vascular access placement.

## Methods

### Study Population

EQUAL study is an ongoing multicenter prospective cohort study among European nephrology-referred patients ≥65 years.^19^ Patients were included in Germany, Italy, the Netherlands, Poland, Sweden, and the UK from 2012 on, when their eGFR first dropped below 20 ml/min per 1.73 m^2^, and were followed for 4 to 8 years or up to kidney transplantation. Patients with acute kidney injury or a history of KRT were excluded. Some patients’ kidney function were higher than 20 ml/min per 1.73 m^2^ at study baseline, as eligibility assessment took place earlier. For the current study, we restricted inclusion to patients with an eGFR between 10 and 30 ml/min per 1.73 m^2^ at baseline, because this was considered a clinically relevant population for KRT prediction and conform to the Grams development study. Clinical characteristics and laboratory values were registered every 6 months. All patients gave written informed consent to participate. Overlap between the EQUAL cohort and the European CKD Prognosis Consortium (CKD-PC) cohort on which the Grams model was developed is possible for the Swedish patients included in EQUAL, but highly unlikely because EQUAL inclusion started 3 years after the formation of the CKD-PC cohort.

### Outcomes

The Grams model predicts the risk of KRT initiation, CVD event and death, within 2 and 4 years. These outcomes are predicted in any possible sequence, for instance the risk of experiencing CVD followed by KRT initiation. For the main analysis of the current study, the following combined outcomes were validated: any KRT, any CVD event, death, and no event. In addition, the outcome of death was split into “death without KRT” and “death after initiation of KRT.” These outcomes were considered most clinically relevant and ensured enough events in our data for precise validation. It is important to note that the calculated risks for combined outcomes no longer represent multinomial probabilities because they are not mutually exclusive; patients may experience various combinations of these outcomes. In accordance with the original model, CVD event was defined as a nonfatal stroke, heart failure, myocardial infarction, or coronary revascularization.

### Predictors

Predictors in the Grams model include age, sex, race, history of CVD, current smoking status, systolic blood pressure, diabetes mellitus, eGFR (using the CKD-EPI equation), and urine albumin-to-creatinine ratio (ACR). These predictors were measured at the first study visit. If ACR was missing but protein-to-creatinine ratio was measured, the latter was converted to ACR using equations developed for this purpose.^20^

### Clinical Impact Projection

For assessment of potential clinical utility, we assessed whether decision rules based on a predicted risk or eGFR can identify patients starting KRT within 1 year and, therefore, be useful to ensure timely KRT preparation. A DCA was employed, which illustrates the difference in impact of various decision-rules and prediction models. We assessed the potential clinical utility for the following predefined referral thresholds: a predicted 2-year KRT risk of 20%, 30%, 40% and 50% (based on the Grams model), an eGFR <30, <20 or <15 ml/min per 1.73 m^2^, and the Kidney Foundation’s Kidney Disease Outcomes Quality Initiative suggested guideline of a 2-year KRT risk >50% and/or an eGFR <15 ml/min per 1.73 m^2^.^16^, ^17^, ^18^ Every patient was assessed at the first study visit and we determined whether their predicted risk or eGFR met the different KRT preparation thresholds. More specifically, if patients reached the above mentioned thresholds, we observed whether they initiated KRT within 1 year; if so, the decision-rule was “correct” and AVF referral was appropriate (a true positive). If the patient did not initiate KRT within 1 year, due to slow progression, death or any other cause, the KRT preparation and AVF placement advice was considered unnecessary or “incorrect” at that time-point (a false positive).^16^ By simplifying 2-year risk predictions into a decision rule (yes/no threshold), we could assess whether these predictions can also be used to guide decisions on KRT initiation within 1 year instead of 2 years. Patients who initiate KRT sooner within the 2-year time-frame generally also have a higher predicted risk of KRT compared to patients who initiate later within that window.

### Statistical Analysis

Continuous baseline values are presented as means with standard deviations when normally distributed, or as medians with interquartile range. To describe the observed occurrence of outcomes, we used cumulative incidence functions and a stacked cumulative incidence plot to account for censoring and competing events. Missing data were assumed to be missing at random and a 10-fold multiple imputation was performed using the R-package (R core team 2021, version 3.5.3) “mice.”^21^ All predictors, outcomes and times to outcomes, along with various other patient characteristics were included in the imputation model and results were pooled according to Rubin’s rules.^22^^,^^23^

For every patient, the predicted risks for each outcome were calculated using the multinomial formulas provided by Grams *et al.*^6^ (these multinomial formulas are also provided in our Supplemental Material). Model performance was assessed by discrimination and calibration. The model performance differs per predicted outcome and outcome grouping and was assessed separately for the various combined outcomes and original outcome trajectories. Discrimination determines how well a model can discriminate between patients who will have the outcome and those who will not and was calculated using a time-to-event C-statistic.^24^ Harrel’s C-statistic was modified to account for competing events using the method proposed by Wolbers *et* *al.*^25^ Calibration determines how well the absolute predicted risk corresponds with the observed risk, overall and in patient subgroups.^24^ Observed risks were calculated using cumulative incidence functions, to take competing events and censoring into account. These cumulative incidences are not the same as multinomial probabilities, which would ideally have been used had follow-up been complete.^26^ The calibration-in-the-large and calibration plots, including a smoothed locally weighted scatterplot smoothing line, were computed.^27^^,^^28^

DCA was used to evaluate the potential clinical utility. This is a statistical method in which the relationship between harm-benefit ratios (also termed threshold probabilities) and the net benefit is plotted to ascertain the added value of different decision rules over the entire range of theoretical harm-benefit ratios (x-axis). The net benefit (y-axis) is calculated by subtracting the proportion of all patients who are false positive (unjustified AVF referral) from the proportion who are true-positive (correct AVF referral), weighting by each theoretical harm-benefit ratio.^29^^,^^30^ For a low harm-benefit ratio, a false positive is given less weight compared to a higher harm-benefit ratio. The harm-benefit ratio is not set or calculated nor does the DCA tell us what the “correct” harm-benefit ratio is; the DCA plots the net benefit for all possible harm-benefit ratio’s, starting at a scenario in which AVF referral and placement is only ever beneficial (a harm-benefit ratio which is almost zero) and ending at a scenario in which an AVF referral is far more harmful than beneficial. When comparing different referral guidelines, the guideline with the highest net benefit on the y-axis across the range of harm-benefit ratios would be considered most beneficial. The DCA is most useful for comparing net benefit of various decision threshold over a wide range of harm-benefit ratios. A more in-depth explanation of the DCA is included in the Supplemental Material (Decision curve analysis). The sensitivity, specificity, positive predictive value and negative predictive value were also calculated for each decision rule.

### Sensitivity Analyses

As a sensitivity analysis, model performance was assessed for all 8 outcome sequences that the Grams model predicts, namely KRT initiation only, KRT initiation after CVD, CVD only, CVD after KRT initiation, death only, death after KRT initiation, death after CVD, and death after KRT initiation and CVD. To assess discrimination of these outcomes, areas under the receiver operating curve were computed and calibration was determined with absolute proportions. Time-to-event and competing risks could not be considered, because the predicted outcomes of the Grams model consist of a series of events and time-points and not all outcomes are competing with each other. We also compared potential clinical impact of the Grams model to the 2-year kidney failure risk equation (KFRE), an often used prediction model for KRT initiation, using DCA.^31^, ^32^, ^33^

This study was performed according to the transparent reporting of a multivariable prediction model for individual prognosis or diagnosis guidelines (see Supplementary Material for TRIPOD checklist).^34^ Analyses were performed in R version 3.5.3.

## Results

### Baseline Characteristics

In total, 1517 patients from EQUAL had an eGFR between 10 ml/min per 1.73 m^2^ and 30 ml/min per 1.73 m^2^ and were included in the study. The baseline characteristics of the EQUAL cohort used for validation are depicted in Table 1, and are compared to the reported characteristics of the development cohort of the Grams model: the CKD-PC cohort.^6^ Compared to the CKD-PC cohort, the median age of patients from the EQUAL cohort was 4 years older. EQUAL included less black patients than the CKD-PC cohort (1.3% vs. 9.3%). EQUAL patients had worse kidney function compared to CKD-PC patients, shown by a lower eGFR and higher ACR.

**Table 1:** Baseline characteristics, compared to the baseline characteristic of the Grams development cohort

Characteristics	EQUAL cohort (*N* = 1517)	Missing %	CKD-PC development cohort^6^
Age (yr)	76 (71–82)	0	72
Sex (% male)	66%	0	61%
Race (% black)	1.3%	0.1	9.3%
Country of residence		0	NR
United Kingdom	30.1%		
Italy	23.2%		
Sweden	18.2%		
The Netherlands	15.4%		
Germany	7.8%		
Poland	5.3%		
History of CVD	39.7%	0	45.1%
Diabetes mellitus	42.3%	1.0	46.2%
Hypertension	89.2%	3.7	NR
SBP (mmHg)	143 (22)	1.6	130
Current smoker	9.0%	19.7	NR
eGFR (ml/min/1.73 m^2^)	18 (4)	0	24
Median uACR (mg/g)	391 (57–1566)	40.3	85^a^
BMI (kg/m^2^)	28 (5)	7.3	NR
Primary kidney disease		16.7	NR
Hypertension	44.3%		
Diabetes mellitus	24.1%		
Glomerular disease	11.2%		
Tubulo-interstitial disease	9.6%		
Other	10.9%		

ACR, albumin-to-creatinine ratio; BMI, body mass index; CKD-PC, chronic kidney disease prognosis consortium; CVD, cardiovascular disease; eGFR, estimated glomerular filtration rate; NR, not reported; SBP, systolic blood pressure; uACR, urine albumin-to-creatinine ratio.

For continuous variables the median or mean is reported with corresponding interquartile range or standard deviation (depending on whether the variable was normally distributed).

a The median of the overall CKD-PC cohort is calculated by taking the mean of all cohort-specific medians and can therefore deviate from the true median.

### Follow-Up and Outcome Assessment

**Figure 1:**
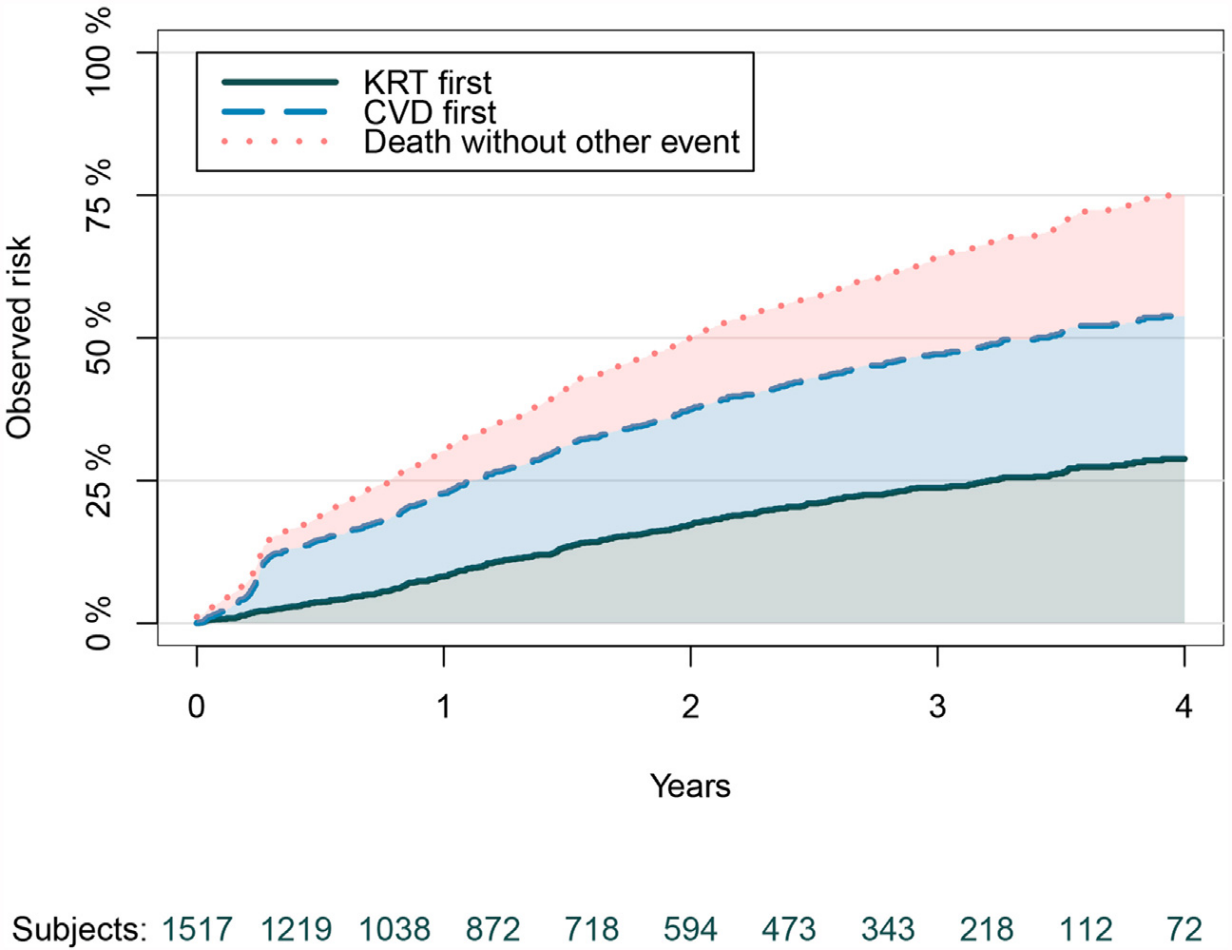
Stacked cumulative incidence plot. The observed incidence of experiencing KRT, CVD or death as first event is shown. Censoring is accounted for and all outcomes are competing events. After 4 years 74.9% of patients experienced a first event of which 28.8% experienced KRT first, 25% CVD first and 21.1% died without experiencing another event. The number of patients remaining in the study at each time-point are shown below the x-axis. CVD, cardiovascular disease; KRT, kidney replacement therapy.

Patients included in the study were followed up for a maximum of 6 years, with an average follow-up time of 2.3 years. Within 2 years, a total of 298 (19.6%) patients started KRT, 310 (20.4%) got a CVD event and 310 (20.4%) died (A patient could experience multiple outcomes). Within 4 years, 426 (28.1%) patients initiated KRT, 365 (24.1%) experienced a CVD event and 493 (32.5%) died (See Supplementary Figure S1 for a flowchart and proportions of all outcomes). The competing risks of experiencing a first event are shown in Figure 1; the probability of not experiencing any event was 50% within 2 years and 25% within 4 years. Among the patients who died within 2 years, the large majority died without starting KRT (80.0% and 72.6% for 2 and 4 year horizons, respectively). Among the patients receiving KRT within 4 years, 72.7% started on HD, 23.5% on peritoneal dialysis and 3.8% received a pre-emptive transplantation. Out of the 310 patients starting HD, 45.4% started with a central venous catheter and 53.2% with a graft or fistula, and 1.4% with unknown vascular access type. These percentages were similar for patients starting KRT within 2 years.

### Predictive Performance

**Figure 2:**
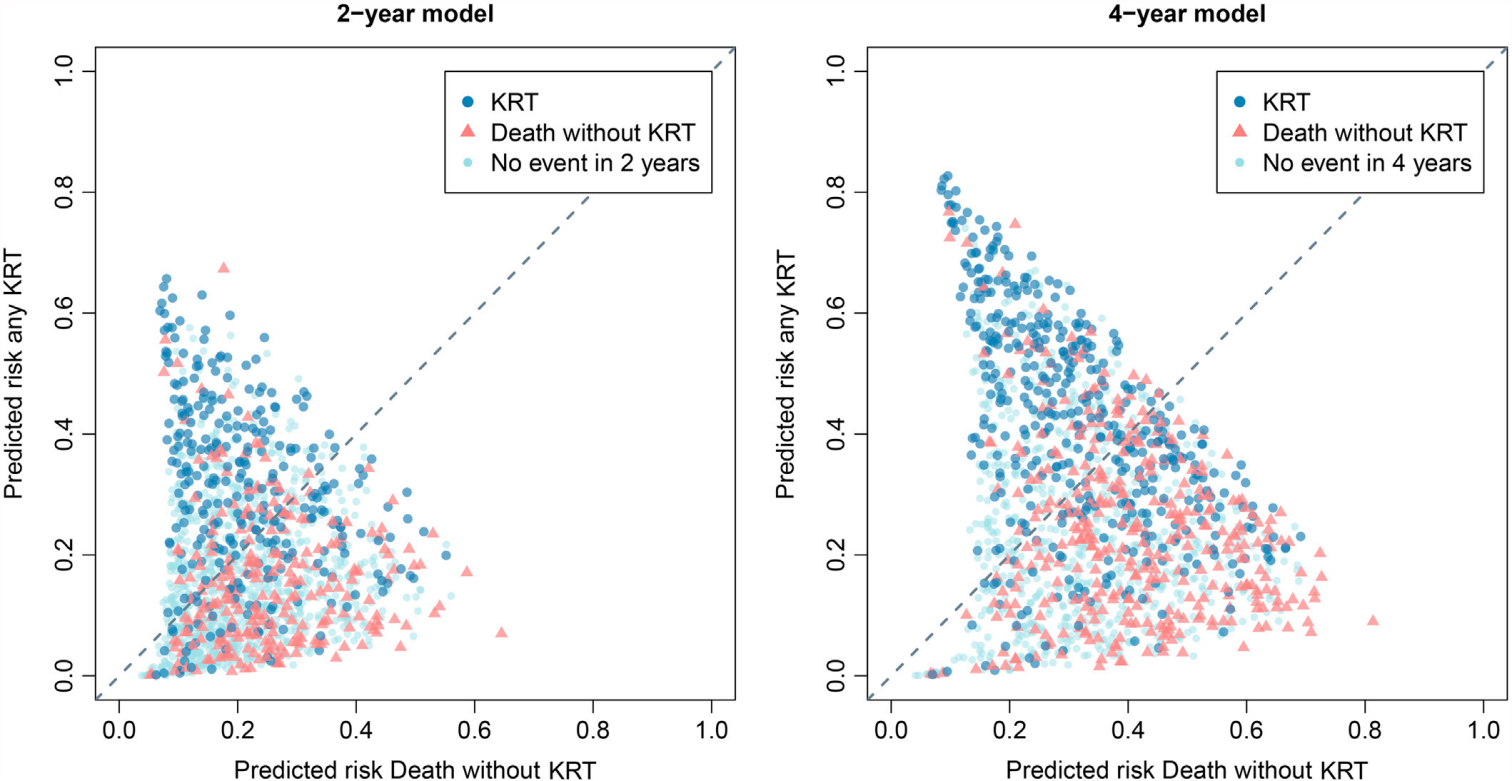
Scatterplot depicting predicted probabilities of KRT against predicted probabilities of death without KRT. Each patients actual observed outcome (death first, KRT or neither) is illustrated by the color and shape of the point. The dotted 45° line indicates where the predicted risks of KRT and death without KRT are equal: above this line patients had a higher predicted risk of KRT compared to death without KRT. KRT, kidney replacement therapy.

**Figure 3:**
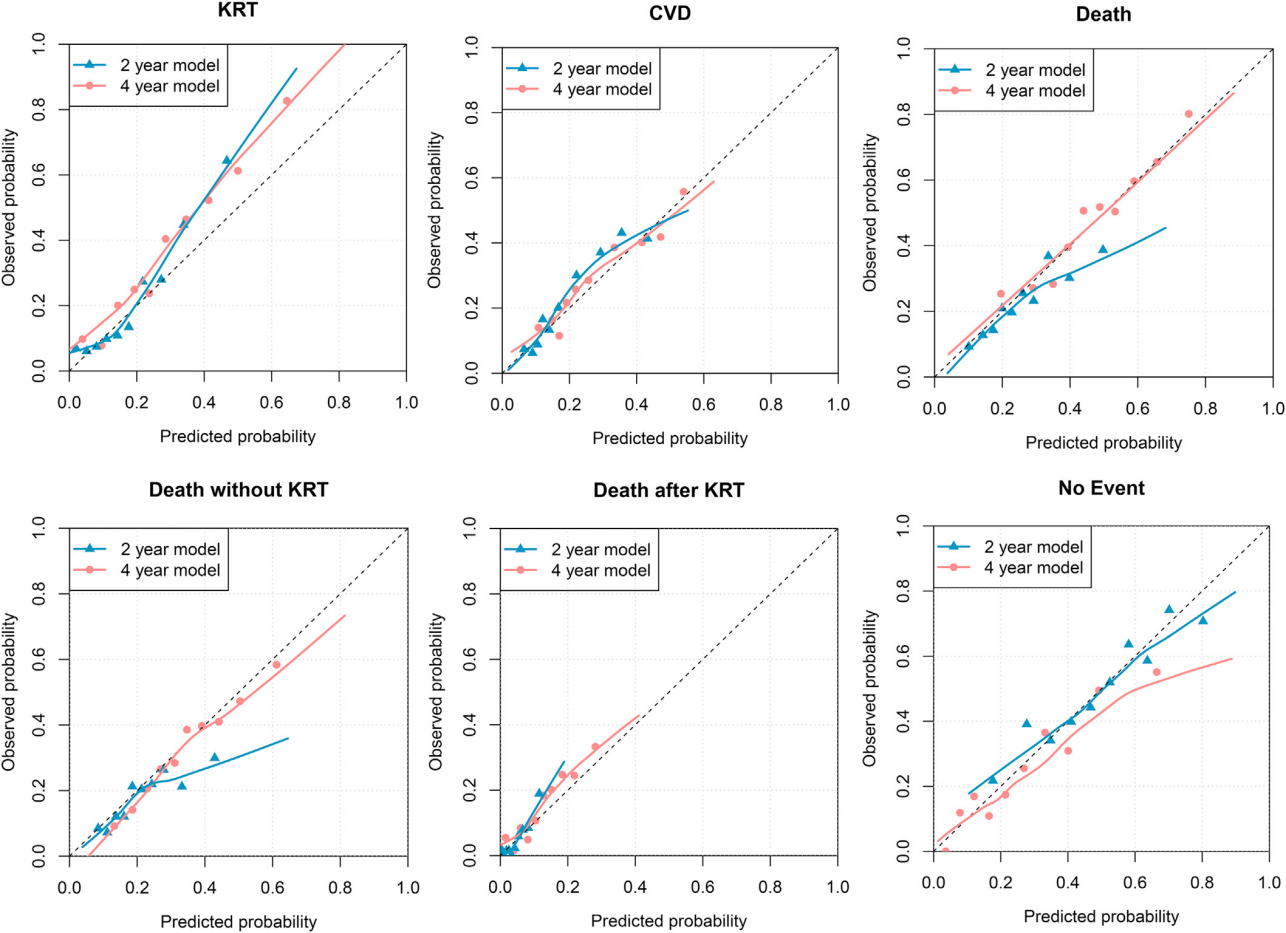
Calibration plots for each main outcome. The predicted probability is shown on the x-axis and the observed outcome rate (calculated with cumulative incidence functions) is given on the y-axis. The dotted 45 degree line represents perfect agreement between predicted and observed probability. The points represent a decile of the validation population (10%), ranked by predicted probability. CVD, cardiovascular disease; KRT,kidney replacement therapy.

The discrimination of the Grams model varied greatly between predicted outcomes and the C-statistics ranged from 0.59 to 0.76 (see Table 2). The best discrimination was seen for predicting KRT (2-year C-statistic 0.74, 4-year C-statistic 0.73) and “Death after KRT” (2-year C-statistic 0.76, 4-year C-statistic 0.72). The discriminative power for predicting no event and overall death was rather poor. The 2-year 4 variable KFRE showed a similar C-statistic of 0.73 (95% confidence interval: 0.70–0.76) for predicting KRT. The scatterplots shown in Figure 2 illustrate the model’s capability of discriminating between patients who will start KRT and those who will die without having started KRT. Though there is some overlap, most patients who died without KRT had a higher predicted risk of death than those with KRT and vice versa. Overall, the calibration of the model was fairly accurate (see Table 2 and Figure 3). For high-risk patients, the model underpredicted the risk of KRT. In general, the 4-year model had better calibration than the 2-year model. The risk of CVD, death, and “death without KRT” were particularly well calibrated in the 4-year model. The discrimination and calibration for all outcome sequences predicted by the Grams model is shown in Supplementary Table S1 and S2 and Supplementary Figure S2. In general, the discriminative ability and calibration of the 2-year and 4-year model ranged from moderate to good for the specific outcomes (area under the receiver operating characteristic curve -AUC- range 0.61–0.79), but due to few events these estimates are rather uncertain.

**Table 2:** External validation, discrimination and calibration-in-the-large results for the 2-year and 4-year Grams model

	2-year model		4-year model	
Outcome	C-statistic (95% CI)	Calibration-in-the-large (predicted vs. observed)	C-statistic (95% CI)	Calibration-in-the-large (predicted vs. observed)
Any KRT	0.741 (0.710–0.772)	18.7% vs. 21.9%	0.727 (0.700–0.754)	28.9% vs. 37.8%
Any CVD	0.703 (0.674–0.732)	19.9% vs. 22.3%	0.689 (0.661–0.717)	28.6% vs. 29.3%
Any death	0.614 (0.582–0.645)	26.2% vs. 23.0%	0.615 (0.589–0.642)	46.8% vs. 48.3%
Death without KRT	0.615 (0.579–0.650)	21.7% vs. 18.0%	0.640 (0.611–0.670)	34.2% vs. 32.1%
Death after KRT	0.757 (0.692–0.822)	4.5% vs. 4.7%	0.723 (0.677–0.769)	12.6% vs. 15.5%
No event	0.588 (0.567–0.609)	49.4% vs. 50.0%	0.605 (0.581–0.630)	27.8% vs. 25.0%

CI, confidence interval; C-statistic, concordance statistic; CVD, cardiovascular disease event; KRT, kidney replacement therapy.

### Clinical Utility and Projected Impact

**Figure 4:**
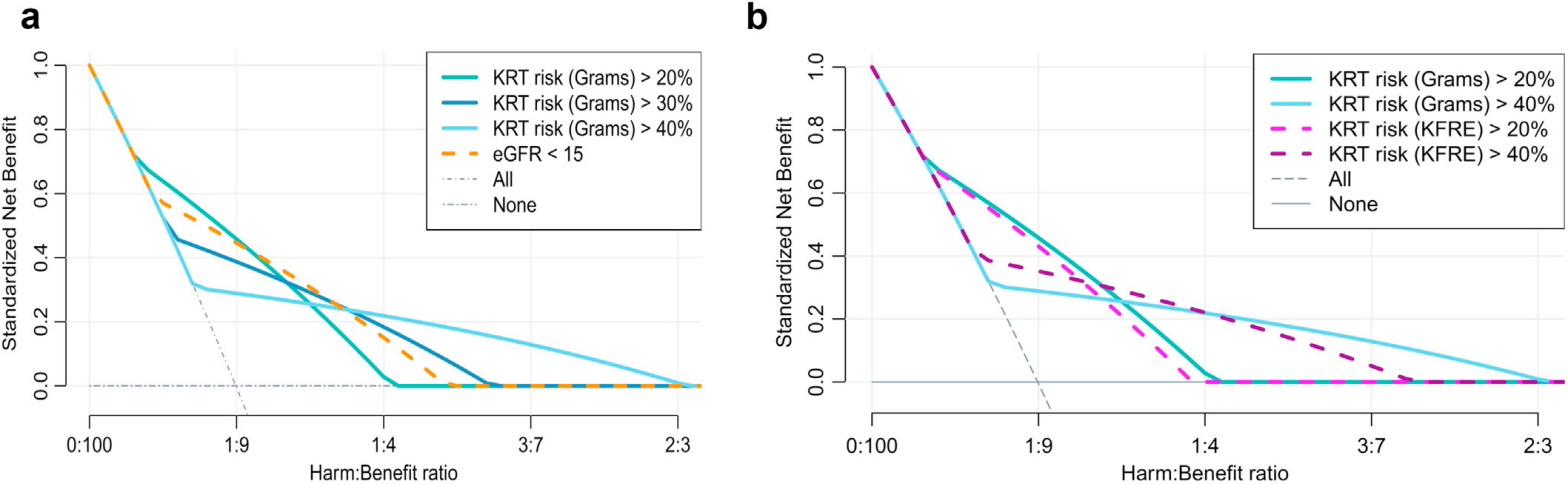
Decision curves showing the clinical utility of the Grams model predictions, eGFR guidelines (a) and KFRE predictions (b) for KRT preparation. KRT preparation (including vascular access referral) is considered appropriate if patients initiate KRT within 1 year. These graphs should be read vertically; for any given harm-benefit ratio the guideline with the highest net benefit would result in the most beneficial ratio of correct referrals and incorrect referrals (given the weight that is given to a false positive compared to a true positive based on the harm-benefit ratio). For most harm-benefit ranges 2-year KRT risks predicted by the Grams prediction model have a higher net benefit than eGFR-based risks (a), the net benefit of the Grams and KFRE predictions are very similar, though the Grams model seems to be slightly more beneficial (b). eGFR, estimated glomerular filteration rate; KRT, kidney replacement therapy.

In Figure 4a and 4b are shown the net benefit of using the Grams model to guide KRT preparations compared to an eGFR referral guideline and compared to the KFRE predictions. The decision curves should be read vertically, meaning that for any given harm-benefit ratio (x-axis) the guideline with the highest net benefit is most beneficial to patients and will result in the most beneficial ratio of justified and unnecessary referrals. If we believe all referrals to be completely harmless, the harm-benefit ratio is very small and the best strategy would be to refer all patients for KRT preparations, including AVF placement. The appropriate harm-benefit ratio will differ for each patient and setting, because a patient’s personal concerns and priorities play a part as well as individual characteristics, which increase the risk of complications surrounding different vascular access forms.^35^^,^^36^ It is therefore more relevant to look at overall superiority of a particular guideline or model, rather than the specific harm-benefit ratios. From Figure 4 we can see that use of the Grams prediction model to guide referral is mostly superior to using the eGFR <15 and results in fewer unnecessary referrals. In Supplementary Figure S3, more eGFR referral thresholds are included in the DCA curve, which clearly demonstrates that eGFR <15 gives a higher net benefit compared to other eGFR thresholds. For situations in which the harm-benefit ratio is small (little harm is expected), a KRT risk >20 % shows the best net benefit; for a medium harm-benefit ratio a decision threshold of eGFR <15 ml/min per 1.73 m^2^ is superior; and in a setting in which the harm could be large, a KRT risk >40% has the highest net benefit. Which of these decision rules should be followed depends on the subjective estimate of the risk-benefit ratio for each individual patient. Using the KFRE instead of the Grams model gave very similar net benefit values. The Kidney Foundation’s Kidney Disease Outcomes Quality Initiative recommended guideline of a 2-year KRT risk ≥ 50% and/or eGFR ≤15 ml/min per 1.73 m^2^ was almost identical to adhering to an eGFR ≤15 ml/min per 1.73 m^2^ guideline (Supplementary Figure S3).

In addition to the net benefit, several impact measures such as the sensitivity and specificity were calculated for the discussed guidelines and thresholds (Table 3). Compared to using eGFR-based guidelines, using risk thresholds would yield notably less referrals and considerably fewer unnecessary referrals (false positives). The combined guideline of 2-year KRT risk >50% and eGFR <15 ml/min per 1.73 m^2^ would only yield 4 more referrals compared to simply referring all patients with an eGFR <15 ml/min per 1.73 m^2^ and therefore has no added benefit over the simple eGFR guideline in our cohort of older patients. The number of false negatives is difficult to directly relate to clinical practice, because these patients may still be referred in time (according to the risk thresholds) at any subsequent doctor’s visit. Similarly, the negative effects of a false positive may also be mitigated by delaying or deferring KRT preparations at subsequent clinical assessments.

**Table 3:** Diagnostic properties for various guidelines that may be used to refer patients for AVF formation. A referral is seen as appropriate (true positive) if KRT is initiated within 1 year

Guidelines	Total referrals	True positives	False positives	True negatives	False negatives	Sensitivity (95% CI)	Specificity (95% CI)	PPV (95% CI)	NPV (95% CI)
eGFR <30 (refer all)	1517	151	1366	0	0	100% (97–100)	0% (0–0)	10% (9–12)	-
eGFR <25	1429	150	1279	87	1	99% (96–100)	6% (5–8)	11% (9–12)	99% (93–100)
eGFR <20	1123	146	977	389	5	97% (92–99)	29% (26–31)	13% (11–15)	99% (97–100)
eGFR <15	424	103	321	1045	48	68% (60–75)	77% (74–79)	24% (20–29)	96% (94–97)
2-yr KRT risk >20%	588	121	467	899	30	80% (73–86)	66% (63–68)	21% (17–24)	97% (95–98)
2-yr KRT risk >30%	304	83	221	1145	68	55% (47–63)	84% (82–86)	27% (22–33)	94% (93–96)
2-yr KRT risk >40%	128	52	76	1290	99	34% (27–43)	94% (93–96)	41% (32–50)	93% (91–94)
2-yr KRT risk >50%	46	21	25	1341	130	14% (9–21)	98% (97–99)	46% (31–61)	91% (90–93)
2-yr KRT risk >50% or eGFR<15	428	103	325	1041	48	68% (60–75)	76% (74–78)	24% (20–29)	96% (94–97)

CI, confidence interval; eGFR, estimated glomerular filtration rate in ml/min/1.73m^2^; KRT, kidney replacement therapy; NPV, negative predictive value; PPV, positive predictive value.

Total referrals are the number of patients that would be referred for AVF formation according to each guideline.

## Discussion

In this study, we externally validated the Grams model in a European, older cohort of nephrology-referred patients with CKD stage 4. Model performance was assessed for the risk of KRT, CVD, and death within 2 years and 4 years. The discriminative ability was reasonably good for predicting KRT but poorer for the outcomes CVD and death. Overall, the calibration was accurate, especially for the 4 year model. The potential clinical utility for decision making regarding KRT preparation (in particular vascular access placement) was investigated and compared to existing eGFR guidelines. Using the model to guide KRT preparation may be more effective than following the currently recommended eGFR thresholds. Depending on a patient’s and their nephrologist’s preferences and circumstances, preparation could be considered at a predicted KRT risk of more than 20%, more than 40% or an eGFR <15.

Considering results from previous studies, the prediction of death is generally less accurate than predicting KRT initiation.^37^ In previous French validation studies, the Grams model was externally validated in a cohort of advanced CKD patients aged 75 years and older for the outcomes KRT and “death without KRT.”^7^^,^^8^ These studies reported a C-statistic of 0.64 and 0.65 for KRT and a C-statistic of 0.68 and 0.70 for “death without KRT” for the 2-year and 4-year models, respectively. Our DCA showed that, for most patients, using individualized risk thresholds has a higher potential clinical utility than using eGFR thresholds, though the eGFR <15 guideline also showed good performance. Particularly in older populations it is important not to solely rely on eGFR, because progression of kidney disease is often slow. Previous literature has also shown that relying on eGFR-based guidelines in older patients would lead to a high proportion of unnecessary vascular access referrals.^38^^,^^39^

This study has a number of strengths. To our knowledge, this is the first study to externally validate the Grams model’s predictive performance for all outcomes. We accounted for censoring and competing risks in the external validation. Another strength is the use of data from a current, international European cohort, which allows generalization of our results to various European countries. Moreover, we examined the potential clinical utility of using the model for decision making regarding KRT preparation, which is an important and often overlooked step toward implementation.^29^^,^^40^ We explored the utility of multiple decision rules over a range of harm-benefit ratios. Our results should be seen in the light of a number of limitations. First, KRT only included patients who initiated dialysis or were transplanted (conforms to the definition used by Grams *et al.*^6^). For prospective use of the model, it would be more informative to also include patients who opted for conservative care in the outcome definition. Second, for the DCA and standard diagnostic measures (such as sensitivity and specificity), censoring due to loss to follow-up was not considered. Because these analyses looked at a shorter time frame, this censoring was less of an issue, but nevertheless present. Furthermore, there was a considerable amount of missing baseline values for ACR. Some patients may delay dialysis start to allow time for vascular access maturation, this may have influenced the outcomes of our decision analysis as well. Finally, the clinical utility analysis is only a first step toward assessing the impact of such a model. The DCA only assesses optimal test diagnostic characteristics related to false positives. A change in practice from using eGFR guidelines to prognostic model probabilities would have broader clinical and potentially economic effects. The harm-benefit ratios are subjective and it is unrealistic to determine the appropriate harm-benefit ratio for individual patients in practice. We were only able to compare the clinical utility of risk thresholds to simple eGFR-based guidelines. These are not an accurate representation of current practice, where decisions are often based on more factors than eGFR, such as the rate of renal function decline and the physician’s experience. Furthermore, the prediction model and decision rules were applied at baseline contrary to clinical practice, where the nephrologist is likely to consider whether KRT preparation is appropriate at each follow-up consultation.

This study may have a number of clinical implications. Using a prediction model of KRT risk (such as the Grams model or KFRE) may provide clinicians with an extra tool to improve the timing of KRT preparations, including AVF placement. A 2-year predicted risk threshold of 20% or 40% seemed most beneficial, depending on the expected potential harms and benefits. Using prediction models for this timing may result in fewer patients unnecessarily undergoing vascular access surgery and may result in more patients initiating dialysis on their preferred vascular access type. Currently, a large proportion of patients undergoing pre-emptive vascular access placement will not start dialysis within a year and especially many older patients will never use their vascular access.^41^^,^^42^ A study by Lee *et al.*^11^ showed that in a cohort of patients aged 70 years and older, 33% of patients that received an AVF did not initiate KRT within 2 years. The Kidney Foundation’s Kidney Disease Outcomes Quality Initiative recommended guideline of 2-year KRT risk >50% or eGFR <15 ml/min per 1.73 m^2^ did not have added value compared to only using the eGFR guideline of <15 ml/min per 1.73 m^2^ in our cohort, which performed well. Although the predicted risks could never replace health care professionals, they can aid in decision making by providing objective risk estimates.^43^ Moreover, they are easy to calculate using the existing webtool (http://ckdpcrisk.org/lowgfrevents/). One could also argue that in older patients it may be beneficial to start dialysis with another vascular access type such as an arteriovenous graft or a central catheter. This would greatly decrease the number of unused AVFs but requires a careful consideration of all involved risks and there is currently an randomized controlled trial running to determine the best vascular access strategy in elderly HD patients.^44^

Future studies may focus on recalibrating the Grams model to predict outcomes within a shorter time frame of 3, 6, 9 or 12 months, because this might be more informative and intuitive to use for decisions regarding KRT preparation. Predicting maturation time of an AVF may further improve this timing. In addition, it would be valuable to externally validate the Grams model in other populations such as younger and non-European patients as well. To simulate practice more closely, it is important to validate such models at every subsequent doctor visit for which a dynamic model may be more fitting. Finally, a clinical impact trial, in which physicians or patients are randomized to the use of a prediction model to augment decision-making will give the most insight into the clinical impact of these models and allow for a comprehensive comparison with current practice, in which many factors besides eGFR are taken into account.

In conclusion, this study provided a first external validation of the complete Grams model. The clinical utility of this model for the timing of vascular access placement was generally superior to existing eGFR guidelines and has potential in combating unnecessary vascular surgeries.

## Disclosure

All the authors declared no competing interests.

## Appendix

### List of EQUAL study investigators are listed in the appendix

Adamasco Cupisti IT, Adelia Sagliocca IT, Alberto Ferraro IT, Aleksandra Musiała PL, Alessandra Mele IT, Alessandro Naticchia IT, Alex Còsaro IT, Alistair Woodman UK, Andrea Ranghino IT, Andrea Stucchi IT, Andreas Jonsson SE, Andreas Schneider DE, Angelo Pignataro IT, Anita Schrander NL, Anke Torp DE, Anna McKeever UK, Anna Szymczak PL, Anna-Lena Blom SE, Antonella De Blasio IT, Antonello Pani IT, Aris Tsalouichos IT, Asad Ullah UK, Barbara McLaren UK, Bastiaan van Dam NL, Beate Iwig DE, Bellasi Antonio IT, Biagio Raffaele Di Iorio IT, Björn Rogland SE, Boris Perras DE, Butti Alessandra IT, Camille Harron UK, Carin Wallquist SE, Carl Siegert NL, Carla Barrett UK, Carlo Gaillard NL, Carlo Garofalo IT, Cataldo Abaterusso IT, Charles Beerenhout NL, Charlotte O'Toole UK, Chiara Somma IT, Christian Marx DE, Christina Summersgill UK, Christof Blaser DE, Claudia D'alessandro IT, Claudia Emde DE, Claudia Zullo IT, Claudio Pozzi IT, Colin Geddes UK, Cornelis Verburgh NL, Daniela Bergamo IT, Daniele Ciurlino IT, Daria Motta IT, Deborah Glowski UK, Deborah McGlynn UK, Denes Vargas SE, Detlef Krieter DE, Domenico Russo IT, Dunja Fuchs DE, Dympna Sands UK, Ellen Hoogeveen NL, Ellen Irmler DE, Emöke Dimény SE, Enrico Favaro IT, Eva Platen DE, Ewelina Olczyk PL, Ewout Hoorn NL, Federica Vigotti IT, Ferruccio Ansali IT, Ferruccio Conte IT, Francesca Cianciotta IT, Francesca Giacchino IT, Francesco Cappellaio IT, Francesco Pizzarelli IT, Fredrik Sundelin SE, Fredrik Uhlin SE, Gaetano Greco IT, Geena Roy UK, Gaetana Porto IT, Giada Bigatti IT, Giancarlo Marinangeli IT, Gianfranca Cabiddu IT, Gillian Hirst UK, Giordano Fumagalli IT, Giorgia Caloro IT, Giorgina Piccoli IT, Giovanbattista Capasso IT, Giovanni Gambaro IT, Giuliana Tognarelli IT, Giuseppe Bonforte IT, Giuseppe Conte IT, Giuseppe Toscano IT, Goffredo Del Rosso IT, Gunilla Welander SE, Hanna Augustyniak-Bartosik PL, Hans Boots NL, Hans Schmidt-Gürtler DE, Hayley King UK, Helen McNally UK, Hendrik Schlee DE, Henk Boom NL, Holger Naujoks DE, Houda Masri-Senghor UK, Hugh Murtagh UK, Hugh Rayner UK, Ilona Miśkowiec-Wiśniewska PL, Ines Schlee DE, Irene Capizzi IT, Isabel Bascaran Hernandez SE, Ivano Baragetti IT, Jacek Manitius PL, Jane Turner UK, Jan-Willem Eijgenraam NL, Jeroen Kooman NL, Joachim Beige DE, Joanna Pondel PL, Joanne Wilcox UK, Jocelyn Berdeprado UK, Jochen Röthele DE, Jonathan Wong UK, Joris Rotmans NL, Joyce Banda UK, Justyna Mazur DE, Kai Hahn DE, Kamila Jędrzejak PL, Katarzyna Nowańska PL, Katja Blouin DE, Katrin Neumeier DE, Kirsteen Jones UK, Kirsten Anding-Rost DE, Knut-Christian Gröntoft SE, Lamberto Oldrizzi IT, Lesley Haydock UK, Liffert Vogt NL, Lily Wilkinson UK, Loreto Gesualdo IT, Lothar Schramm DE, Luigi Biancone IT, Łukasz Nowak PL, Maarten Raasveld NL, Magdalena Durlik PL, Manuela Magnano IT, Marc Vervloet NL, Marco Ricardi IT, Margaret Carmody UK, Maria Di Bari IT, Maria Laudato IT, Maria Luisa Sirico IT, Maria Stendahl SE, Maria Svensson SE, Maria Weetman UK, Marjolijn van Buren NL, Martin Joinson UK, Martina Ferraresi IT, Mary Dutton UK, Merel van Diepen NL, Michael Matthews UK, Michele Provenzano IT, Monika Hopf DE, Moreno Malaguti IT, Nadja Wuttke DE, Neal Morgan UK, Nicola Palmieri IT, Nikolaus Frischmuth DE, Nina Bleakley UK, Paola Murrone IT, Paul Cockwell UK, Paul Leurs NL, Paul Roderick UK, Pauline Voskamp NL, Pavlos Kashioulis SE, Pawlos Ichtiaris DE, Peter Blankestijn NL, Petra Kirste DE, Petra Schulz DE, Phil Mason UK, Philip Kalra UK, Pietro Cirillo IT, Pietro Dattolo IT, Pina Acampora IT, Rincy Sajith UK, Rita Nigro IT, Roberto Boero IT, Roberto Scarpioni IT, Rosa Sicoli IT, Rosella Malandra IT, Sabine Aign DE, Sabine Cäsar DE, Sadie van Esch NL, Sally Chapman UK, Sandra Biribauer DE, Santee Navjee UK, Sarah Crosbie UK, Sharon Brown UK, Sheila Tickle UK, Sherin Manan DE, Silke Röser DE, Silvana Savoldi IT, Silvio Bertoli IT, Silvio Borrelli IT, Siska Boorsma NL, Stefan Heidenreich DE, Stefan Melander SE, Stefania Maxia IT, Stefano Maffei IT, Stefano Mangano IT, Stephanie Palm DE, Stijn Konings NL, Suresh Mathavakkannan UK, Susanne Schwedler DE, Sylke Delrieux DE, Sylvia Renker DE, Sylvia Schättel DE, Szyszkowska Dorota PL, Teresa Cicchetti IT, Teresa Nieszporek PL, Theresa Stephan DE, Thomas Schmiedeke DE, Thomas Weinreich DE, Til Leimbach DE, Tiziana Rappa IT, Tora Almquist SE, Torsten Stövesand DE, Udo Bahner DE, Ulrika Jensen SE, Valentina Palazzo IT, Walter De Simone IT, Wolfgang Seeger DE, Ying Kuan UK, Zbigniew Heleniak PL, Zeynep Aydin NL.

## Supplementary Material

Supplementary File (PDF)

TRIPOD Checklist.

Multinomial formulas for predicted risk calculations.

Decision curve analysis.

**Figure S1.** Inclusion and follow-up flowchart

**Figure S2.** Calibration plots for all possible outcomes

**Figure S3.** Decision Curve comparing the clinical utility of Grams and eGFR thresholds for referring patients to the vascular surgeon for AVF formation

**Table S1.** Discrimination of the 2-year and 4-year Grams model for all possible outcomes

**Table S2.** Calibration of the 2- and 4-year Grams model validated in EQUAL

TRIPOD Checklist.

Multinomial formulas for predicted risk calculations.

Decision curve analysis.
