# Supplementary material for: Predicting Kidney Failure, Cardiovascular Disease and Death in Advanced CKD Patients
Source: Kidney Int Rep. 2022 Aug 2;7(10):2230–41. doi: 10.1016/j.ekir.2022.07.165 (PMC9546766; doi:10.1016/j.ekir.2022.07.165)
Supplement: Supplementary File (PDF) [file mmc1.pdf]

Supplementary Material for:

Predicting kidney failure, cardiovascular disease and death in advanced CKD patients.

Corresponding author: Chava L. Ramspek, c.l.ramspek@lumc.nl

TRIPOD Checklist: Prediction Model Validation

**Section/Topic**		**Checklist Item**	**Page**
**Title and abstract**			
Title		Identify the study as developing and/or validating a multivariable prediction model, the target population, and the outcome to be predicted.	**1**
Abstract		Provide a summary of objectives, study design, setting, participants, sample size, predictors, outcome, statistical analysis, results, and conclusions.	**1**
**Introduction**			
Background and objectives		Explain the medical context (including whether diagnostic or prognostic) and rationale for developing or validating the multivariable prediction model, including references to existing models.	**intro**
		Specify the objectives, including whether the study describes the development or validation of the model or both.	**intro**
**Methods**			
Source of data		Describe the study design or source of data (e.g., randomized trial, cohort, or registry data), separately for the development and validation data sets, if applicable.	**methods**
		Specify the key study dates, including start of accrual; end of accrual; and, if applicable, end of follow-up.	**methods**
Participants		Specify key elements of the study setting (e.g., primary care, secondary care, general population) including number and location of centres.	**methods**
		Describe eligibility criteria for participants.	**methods**
		Give details of treatments received, if relevant.	**N.A.**
Outcome		Clearly define the outcome that is predicted by the prediction model, including how and when assessed.	**methods**
		Report any actions to blind assessment of the outcome to be predicted.	**N.A.**
Predictors		Clearly define all predictors used in developing or validating the multivariable prediction model, including how and when they were measured.	**methods**

		Report any actions to blind assessment of predictors for the outcome and other predictors.	**N.A.**
Sample size		Explain how the study size was arrived at.	**methods**
Missing data		Describe how missing data were handled (e.g., complete-case analysis, single imputation, multiple imputation) with details of any imputation method.	**methods**
Statistical analysis methods		For validation, describe how the predictions were calculated.	**methods, supplement**
		Specify all measures used to assess model performance and, if relevant, to compare multiple models.	**methods**
		Describe any model updating (e.g., recalibration) arising from the validation, if done.	**N.A.**
Risk groups		Provide details on how risk groups were created, if done.	**N.A.**
Development vs. validation		For validation, identify any differences from the development data in setting, eligibility criteria, outcome, and predictors.	**methods**
**Results**			
Participants		Describe the flow of participants through the study, including the number of participants with and without the outcome and, if applicable, a summary of the follow-up time. A diagram may be helpful.	**results, supplement**
		Describe the characteristics of the participants (basic demographics, clinical features, available predictors), including the number of participants with missing data for predictors and outcome.	**results**
		For validation, show a comparison with the development data of the distribution of important variables (demographics, predictors and outcome).	**results, Table 1**
Model performance		Report performance measures (with CIs) for the prediction model.	**results, Table 2, supplement**
Model-updating		If done, report the results from any model updating (i.e., model specification, model performance).	**N.A.**
**Discussion**			
Limitations		Discuss any limitations of the study (such as nonrepresentative sample, few events per predictor, missing data).	**discussion**
Interpretation		For validation, discuss the results with reference to performance in the development data, and any other validation data.	**N.A.**
		Give an overall interpretation of the results, considering objectives, limitations, results from similar studies, and other relevant evidence.	**discussion**

Implications		Discuss the potential clinical use of the model and implications for future research.	**discussion**
**Other information**			
Supplementary information		Provide information about the availability of supplementary resources, such as study protocol, Web calculator, and data sets.	**Throughout manuscript**
Funding		Give the source of funding and the role of the funders for the present study.	**provided**

Multinomial formulas for predicted risk calculations

Web calculator: http://ckdpcrisk.org/lowgfrevents/
Prognostic indexes (PI) were calculated using regression coefficients provided in the Grams supplementary material Table S7. Herein, variables should be entered as follows: age is centred at 60 years and entered per 10 years. For sex, male is entered as 1, female as 0. For race, black is entered as 1, rest as 0. Systolic blood pressure is centred at 140 mmHg and entered per 20 mmHg. eGFR is centred at 25 mL/min/1.73m^2 and entered per 5 mL/min/1.73m^2. For diabetes, CVD history and smoking status, yes is entered as 1, no as 0.
Probabilities were calculated using the following formula:

$$P(Y_{i=1}) = \frac{e^{PI1}}{1 + \sum e^{PIi}}$$

Probabilities of main outcomes for analysis were calculated by combining specific probabilities:

$$P_{RRT} = P_{RRT\ only} + P_{RRT\ after\ CVD} + P_{CVD\ after\ RRT} + P_{death\ after\ RRT} + P_{death\ after\ RRT\ and\ CVD}$$

$$P_{CVD} = P_{CVD\ only} + P_{RRT\ after\ CVD} + P_{CVD\ after\ RRT} + P_{death\ after\ CVD} + P_{death\ after\ RRT\ and\ CVD}$$

$$P_{death} = P_{death\ only} + P_{death\ after\ RRT} + P_{death\ after\ CVD} + P_{death\ after\ CVD\ and\ RRT}$$

$$P_{death\ wihout\ RRT} = P_{death\ only} + P_{death\ after\ CVD}$$

$$P_{death\ after\ any\ RRT} = P_{death\ after\ RRT} + P_{death\ after\ RRT\ and\ CVD}$$

Decision curve analysis

DCA is a statistical method to calculate the net benefit of using certain decision algorithms, or referral guidelines, by taking into account the harms and benefits of an intervention. The net benefit is calculated for all possible harm:benefit ratio's. In the so-called opt-in method of DCA, the net benefit of using a certain guideline can be interpreted as the benefit of correct referrals minus the weighted harms due to unnecessary referrals. A referral was considered correct when patients initiated RRT within 1 year after baseline. The maximum net benefit is the benefit when referring all patients who will get the outcome within the relevant timeframe and not referring anyone unnecessary, while a net benefit of 0 refers to referring no one. The net benefit is determined using the following formula: Net benefit = (true positives/total patients) – (false positives/total patients) * (p_t/(1-p_t)). Herein, p_t/(1-p_t) equals the harm:benefit ratio. The net benefit of each guideline was standardized by dividing it by the prevalence of the outcome and then plotted against a range of harm:benefit ratio's, yielding the decision curve.

Table S1 – Discrimination of the 2- and 4-year Grams model for all possible outcomes validated in EQUAL.

	2-year model		4-year model	
	**AUC (95% CI)**	**N events**	**AUC (95% CI)**	**N events**
**RRT only**	0.740 (0.696-0.784)	180	0.734 (0.694-0.774)	217
**RRT after CVD**	0.770 (0.694-0.847)	43	0.773 (0.704-0.843)	58
**CVD only**	0.702 (0.660-0.744)	152	0.677 (0.625-0.729)	113
**CVD after RRT**	0.667 (0.484-0.850)	13	0.664 (0.491-0.837)	16
**Death only**	0.613 (0.567-0.658)	174	0.647 (0.609-0.686)	238
**Death after RRT**	0.765 (0.678-0.853)	34	0.707 (0.645-0.769)	77
**Death after CVD**	0.697 (0.636-0.758)	74	0.701 (0.650-0.753)	120
**Death after RRT and CVD**	0.794 (0.694-0.895)	28	0.725 (0.657-0.794)	58

AUC, area under de receiver operating curve; CI, confidence interval; RRT, renal replacement therapy; CVD, cardiovascular disease event.

Table S2 – Calibration of the 2- and 4-year Grams model validated in EQUAL.

	Calibration-in-the-large (predicted vs observed*)	
	**2-year model**	**4-year model**
**RRT only**	12.0% vs. 11.9%	12.9% vs. 14.3%
**RRT after CVD**	0.7% vs. 2.6%	1.0% vs. 3.6%
**CVD only**	10.2% vs. 10.0%	9.1% vs. 7.5%
**CVD after RRT**	1.4% vs. 0.9%	2.5% vs. 1.1%
**Death only**	15.3% vs. 11.5%	21.9% vs. 15.7%
**Death after RRT**	3.4% vs. 2.2%	8.9% vs. 5.1%
**Death after CVD**	6.4% vs. 4.9%	12.4% vs. 7.9%
**Death after RRT and CVD**	1.1% vs. 1.9%	3.7% vs. 3.8%

*Observed risks are proportions of outcomes as observed in the data, not accounting for censoring or competing events. RRT, renal replacement therapy; CVD, cardiovascular disease event.

Figure S1: Inclusion and follow-up flowchart

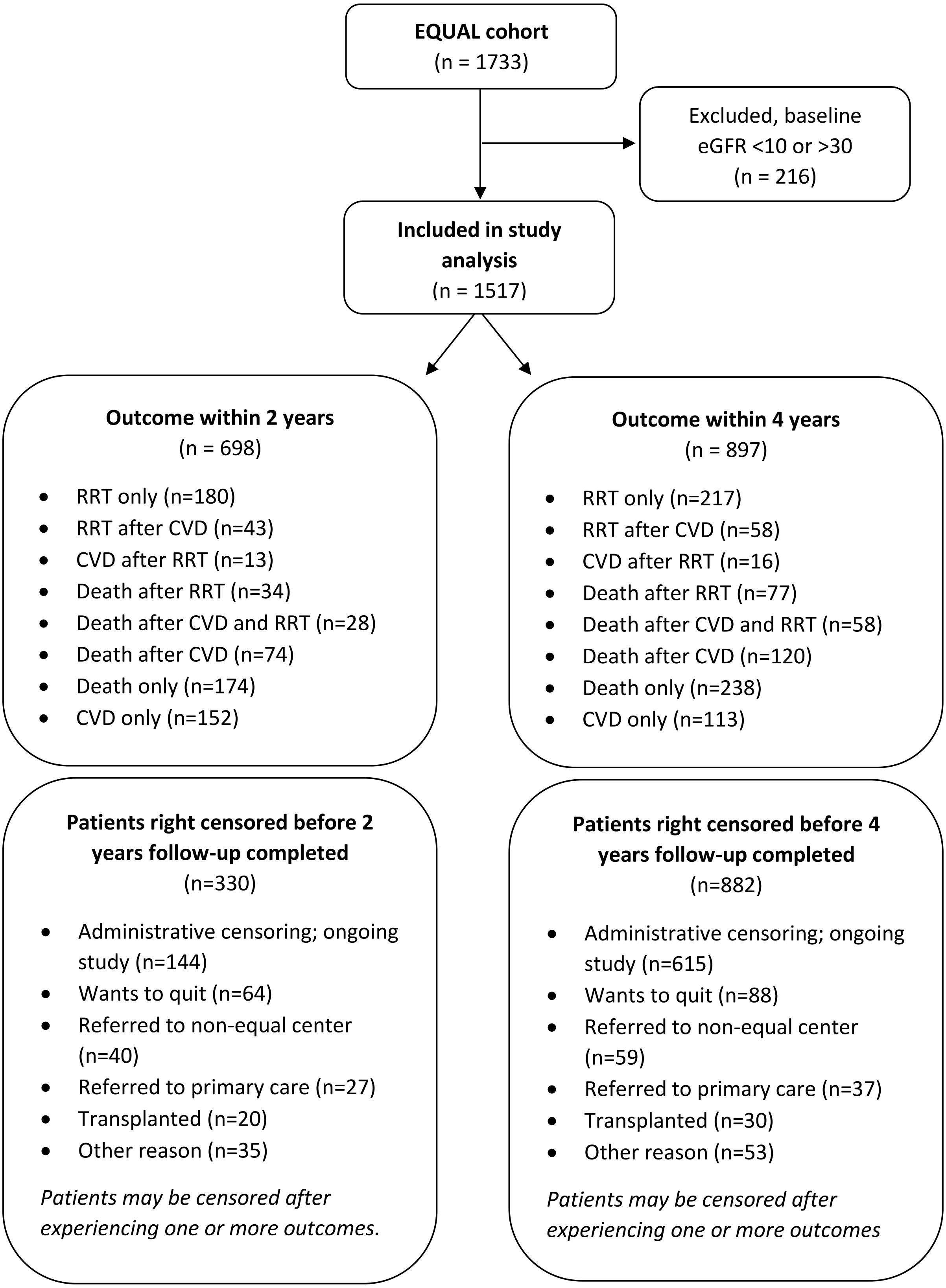

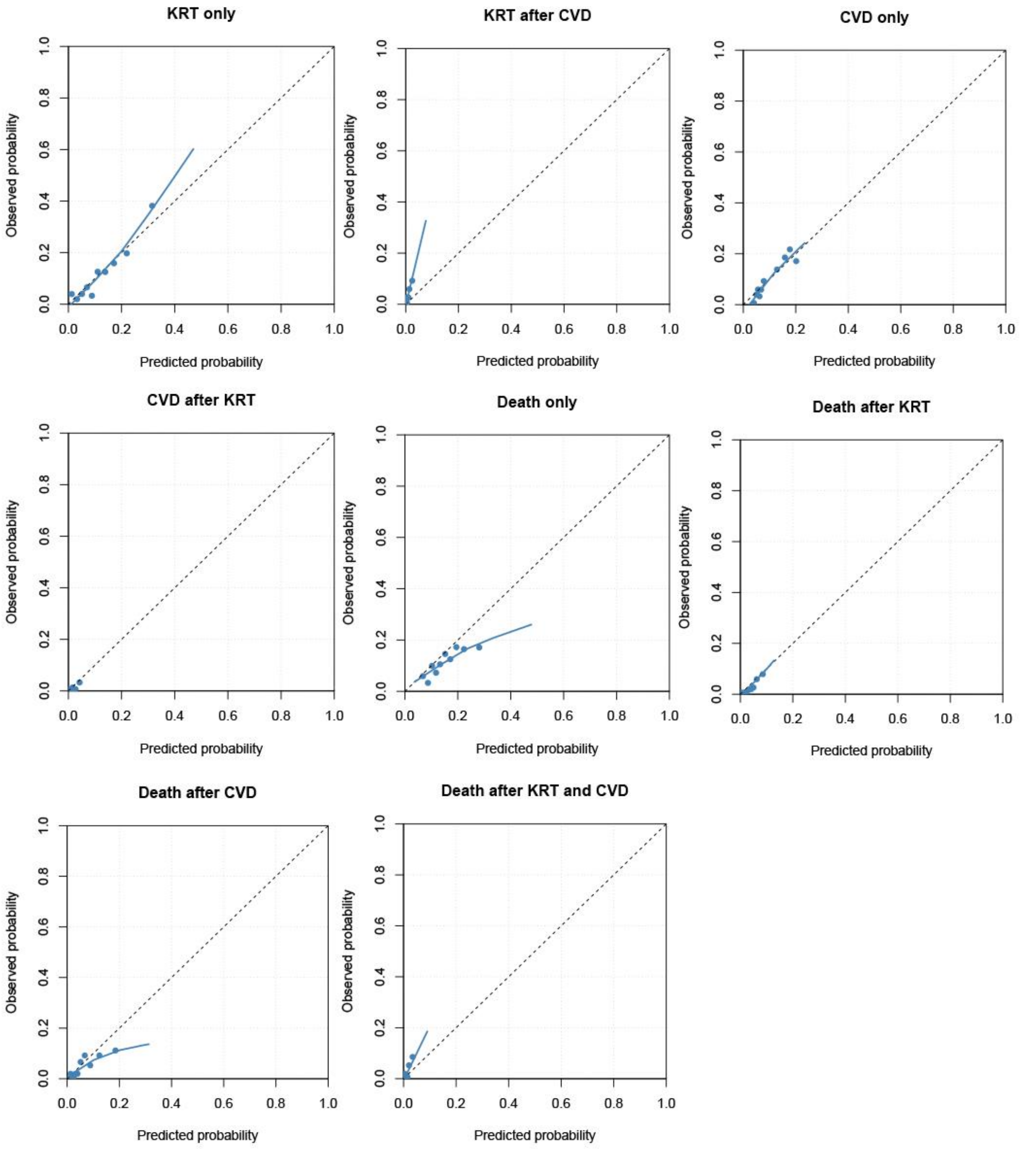

Figure S2 Calibration plots for all possible outcomes.

Observed risks are proportions of outcomes as observed in the data, not accounting for censoring or competing events.

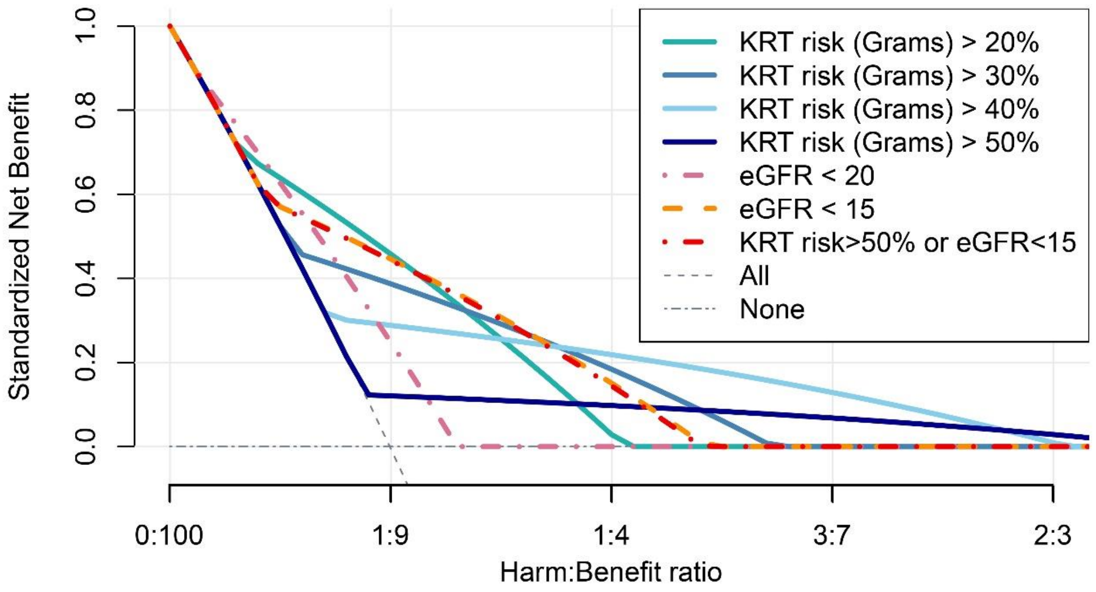

Figure S3: Decision Curve comparing the clinical utility of Grams thresholds and various eGFR based recommendations. A referral is considered appropriate if patients initiate KRT within 1 year. These graphs should be read vertically; for any given harm-benefit ratio the guideline with the highest net benefit would result in the most beneficial ratio of correct referrals and incorrect referrals (given the weight that is given to a false positive compared to a true positive based on the harm-benefit ratio). If a vascular access placement were to be completely harmless (most left point on the x-axis), every patient should be referred to the vascular surgeon. Which harm-benefit ratio is relevant will differ per individual, and is impossible to determine precisely.
